# Epilepsy Combined With Multiple Gene Heterozygous Mutation

**DOI:** 10.3389/fped.2022.763642

**Published:** 2022-03-01

**Authors:** He Qiuju, Zhuang Jianlong, Wen Qi, Li Zhifa, Wang Ding, Sun Xiaofang, Xie Yingjun

**Affiliations:** ^1^Department of Obstetrics and Gynaecology, Key Laboratory for Major Obstetric Diseases of Guangdong Province, The Third Affiliated Hospital of Guangzhou Medical University, Guangzhou, China; ^2^Key Laboratory of Reproduction and Genetics of Guangdong Higher Education Institutes, The Third Affiliated Hospital of Guangzhou Medical University, Guangzhou, China; ^3^Department of Clinical Medicine, The Third Clinical School of Guangzhou Medical University, Guangzhou, China; ^4^Prenatal Diagnosis Center, Quanzhou Women's and Children's Hospital, Quanzhou, China; ^5^Gastrointestinal Surgery, The Third Affiliated Hospital of Guangzhou Medical University, Guangzhou, China

**Keywords:** whole exome sequencing, Sanger sequencing, epilepsy, coexpression analysis, heterozygous mutations

## Abstract

The fast pace of gene discovery has resulted in groundbreaking advances in the field of epilepsy genetics. Clinical testing using comprehensive gene panels, exomes, or genomes is now increasingly available and has significantly increased the diagnostic yield for early-onset epilepsies and enabled precision medicine approaches. In this paper, we report a case of epilepsy in a pedigree. The proband had heterozygous mutations in *KCNC1* (NM_001112741.1:c.959G>A, p. Arg320His), *CAPN3* (NM_000070.2:c.526G>A, p. Val176Met), and *NEFH* (NM_021076.3:c. 2595 delC, p. Lys866Argfs^*^51). Sanger sequencing verification was consistent with the results of whole-exome sequencing. The *KCNC1* mutation was a *de novo* mutation, and the *CAPN3* and *NEFH* mutations were inherited from their father and mother, respectively. Based on the American College of Medical Genetics and Genomics (ACMG) guidelines, a heterozygous mutation was found for *APOB* (NM_000384.2: c.10579C > T, p. Arg3527Trp). The heterozygous mutation at this site was inherent in the pedigree. Coexpression analysis indicated that heterozygous mutations of *KCNC1, CAPN3, NEFH*, and *APOB* were closely related to the clinical phenotypes of the patient, and the clinical phenotypic heterogeneity of the disease may be the result of the interaction of multiple genes.

## Introduction

Epilepsy is a disorder of the central nervous system caused by abnormal discharge of neurons that affects consciousness, sensation, temperament, and movement. The classification levels of epilepsy diagnosis are based on seizure type, epilepsy type (focal, generalized, combined generalized and focal, and unknown) and epilepsy syndrome, and 40% of epilepsy is related to genetic factors (1–4). More than half of active epilepsy cases are 12 years old. The clinical features of epilepsy are complex and diverse and can manifest as paroxysmal movement, abnormal autonomic nerve function, transient sensory disorders, limb convulsions, loss of consciousness, and mental disorders. The etiology of epilepsy is heterogeneous and includes genetic factors, brain diseases, and systemic diseases. An imbalance between excitation and inhibition of the central nervous system leads to epileptic attack, and ion-channel abnormalities are closely related to the occurrence of disease. Mutations in coding genes can participate in the development of the disease by affecting the functioning of ion channels, including those for potassium, calcium, and sodium ions. Approximately 25% of hereditary epilepsy cases are caused by variations in ion channel-related genes (5). In addition, epilepsy is related to changes in neurotransmitters and glial cells (6). Epilepsy can be controlled in most patients through regular medication, but some patients develop refractory epilepsy. Long-term repeated seizures can lead to cognitive impairment, which places a heavy economic and psychological burden on patients and families. Defining the pathogenesis of epilepsy has practical significance for classification, prenatal diagnosis, genetic screening, and subsequent treatment. With the rapid development of high-throughput sequencing technology, whole-exome sequencing technology is playing an important role in the genetic diagnosis of hereditary diseases. Mutations in genetic diseases are mostly located in coding regions, which account for 1% of the whole genome. The whole-exome sequencing technique has the advantages of low cost and strong pertinence. This technique covers most variations associated with gene function (7), enabling screening for pathogenic genes for a variety of diseases (8–11). In the present report, we elucidate the causative role of the interaction of multiple heterozygous mutation genes in the pathogenesis of a severe form of epilepsy.

## Materials and Methods

### Case Presentation

A 26-year-old man came to the hospital because of his recurrent limb convulsions, unstable gait, and ease of falling accompanied by loss of consciousness for more than 5 years. The first seizure occurred at the age of 6 years, and he manifested generalized convulsions, foaming at the mouth, a blue complexion, and non-responsivity. The seizure lasted approximately 2 min. Then, he exhibited no abnormality upon waking, later developed clonic attacks, unintentional movements, and hand and foot paralysis, and received Depakine treatment in the same year (sodium valproate syrup). The next year, the patient experienced large twitching of the legs for 2 min. When the patient discontinued the drug for 2 months, the symptoms were aggravated, characterized by handshaking and eyelid movements, followed by weakness of the legs, trembling of the limbs, and convulsions of the whole body when he was frightened or stressed.

The patient was a first full-term baby and was delivered *via* Cesarean section due to a misplaced position and a large head. The proband was in good condition after birth. Compared with children of the same age, the patient has poor balance function and poor coordination. The patient's parents denied a family history of epilepsy. At the age of 6, the proband underwent a computerized tomography (CT) examination of the brain, and no obvious abnormalities were found. Further electroencephalogram (EEG) and CT examinations were performed, and no obvious abnormalities were found. At the age of 8, the patient had a second seizure, and EEG examination showed inhibition of the α wave. Further 24-h dynamic EEG examination showed abnormal EEG (evident in the right frontotemporal central region, epileptiform discharge) and inhibition of α waves. Based on magnetic resonance imaging (MRI) results, sclerosis of the right hippocampus was suspected. At the age of 9, the proband underwent brain positron emission computed tomography (PET CT), which showed no significant abnormalities in interictal pet metabolic brain imaging and a single spike in the right frontal region during light sleep. At the age of 26, an MRI of the brain showed no significant abnormalities. Multiple external hospital examinations were performed, and no obvious abnormalities in the EEG, MRI, or CT results were found. The muscles were tested more than 10 years ago and have not since. No muscular problems were found_by electromyography (EMG) and nerve conduction velocity (NCV). Recent clinical follow-up shows that a major seizure (violent convulsions) occurred once, in the last 2 years, and myoclonus often occurred.

### Karyotyping Analysis

Peripheral blood was collected intravenously from the proband and his parents, and the peripheral blood lymphocytes were cultured and harvested. A karyotyping analysis was performed according to the conventional G-banding technique (550-band resolution) (12).

### Chromosomal Microarray Analysis

The CytoScan HD chip has a 2 M-probe count and is a high-density chip for detecting copy number variations and single nucleotide polymorphisms. Blood samples of the proband and his parents were collected, and the Affymetrix cyto HD Array was performed in strict accordance with the chip operation manual using CytoScan chip technology provided by Affymetrix.

### Library Preparation and Whole-Exome Sequencing

Venous blood was withdrawn from the proband and his parents, and genomic DNA was extracted according to the manufacturer's standard procedure for the MagPure Buffy Coat DNA Midi KF Kit (Magen, Guangzhou, China). Then, the genomic DNA was fragmented by Segmentase (BGI, China) to generate small DNA fragments (100–500 bp) that were further screened using magnetic beads to enrich the fragments with sizes ranging from 280 to 320 bp. After the ends were filled, an “A” base was added to the 3' end to enable ligation of the DNA fragment to an adapter with a “T” base at the 3' end. The DNA fragments were amplified by a ligation-mediated polymerase chain reaction and purified to form a library. The library was enriched by array hybridization following a protocol (Roche NimbleGen, Madison, USA), followed by elution and postcapture amplification. The magnitude of the enrichment of the products was measured using an Agilent 2100 Bioanalyzer. All the amplified libraries were subsequently sent to BGI for circularization and sequencing on the MGIseq-2000 platform with a paired-end 100 sequencing strategy. The sequenced data were automatically demultiplexed by index.

### Bioinformatics Analysis

We used published filtering criteria to generate “clean reads” for further analysis (13). The “clean reads” were aligned to the human genome reference (hg19) using Burrows Wheeler Aligner (BWA) software (14). The output files were used to perform sequencing coverage and depth analysis of the target region and single-nucleotide variant (SNV), as well as insertion and deletion (indel) calling. We used GATK to detect SNVs and indels. All SNVs and indels were filtered and estimated *via* multiple databases, including dbSNP, HapMap, 1000 human genome datasets and databases of 100 healthy Chinese adults. We used the scale-invariant feature transform (SIFT) and Polyphen2 to predict the effect of variants. Pathogenic variants were assessed under the protocol issued by the American College of Medical Genetics (ACMG) (15). The Human Gene Mutation Database (HGMD) was used to screen for mutations reported in published studies.

### Sanger Sequencing

Mutations in the targeted genes for the proband and his parents were validated using conventional Sanger sequencing methods. Segregation analysis was performed if DNA from family members was available.

### Protein–Protein Interaction Analysis

The protein–protein interactions of targeted genes with the heterozygous mutations confirmed by Sanger sequencing were analyzed with STRING version 10.0 (16) (https://stringdb.org/cgi/input.pl?sessionId=GQHzYg5cCT15&input_page_show_search=on).

## Results

### Karyotyping Analysis and Chromosomal Microarray Analysis

The results of a karyotyping analysis indicated that the karyotypes of the three blood samples were normal. Chromosomal microarray analysis did not identify obvious copy number variations, ruling out the contribution of chromosomal abnormalities and copy number variations to the disease in the family (data not shown).

### Whole-Exome Sequencing Analysis

Whole-exome sequencing identified a new heterozygous mutation c.959G>A (p. Arg320His) in the *KCNC1* gene. *KCNC1* encodes a voltage-gated potassium channel expressed in inhibitory neurons, and mutations in the gene cause progressive myoclonus epilepsy and ataxia. A suspected pathogenic variation consistent with the phenotype of the subject was detected in the *CAPN3* gene associated with limb-band muscular dystrophy type 2A/limb-girdle muscular dystrophy II. Due to the absence of relevance to the pathogenicity of this variant, the mutation was considered a causative mutation according to the ACMG guidelines (15, 17–19). Whole-exome sequencing also detected a suspected pathogenic mutation in the axonal *NEFH* genes related to Charcot-Marie-Tooth disease type 2. There are no reports on the pathogenicity of this mutation (NEFH c.2595delC, p. Lys866Argfs*51). According to the ACMG guidelines, this variation was considered a likely pathogenic variation. In addition, the WES test results indicated a heterozygous mutation (APOB, c.10579C>T, p. Arg3527Trp), and pathogenicity of this variant has been reported (20). According to the ACMG guidelines, the patient had no relevant clinical manifestations, and the mutation was suspected to be a disease-causing variant (Table 1).

**Table 1:** Results of gene mutation in patients with whole exome sequencing.

**Gene**	**Site**	**Coding DNA change**	**Protein change**	**Disease associated with the gene**
*KCNC1*	Chr11: 17793600	NM_001112741.1:c.959G>A	p. Arg320His	Progressive myoclonic epilepsy type 7
*CAPN3*	Chr15: 42679978	NM_000070.2: c.526G>A	p. Val176Met	Limb band muscular dystrophy type 2A/limb girdle muscular dystrophy 11
*NEFH*	Chr22: 29886222	NM_021076.3: c.2595delC	p. Lys866Arg fs*51	Charcot-Marie-Tooth disease type 2 hypercholesterol
*APOB*	Chr2: 21229161	NM_000384.2: c.10579C>T	p. Arg3527Trp	Type 1/autosomal dominant hypercholesterolemia type B

### Sanger Sequencing

**Figure 1:**
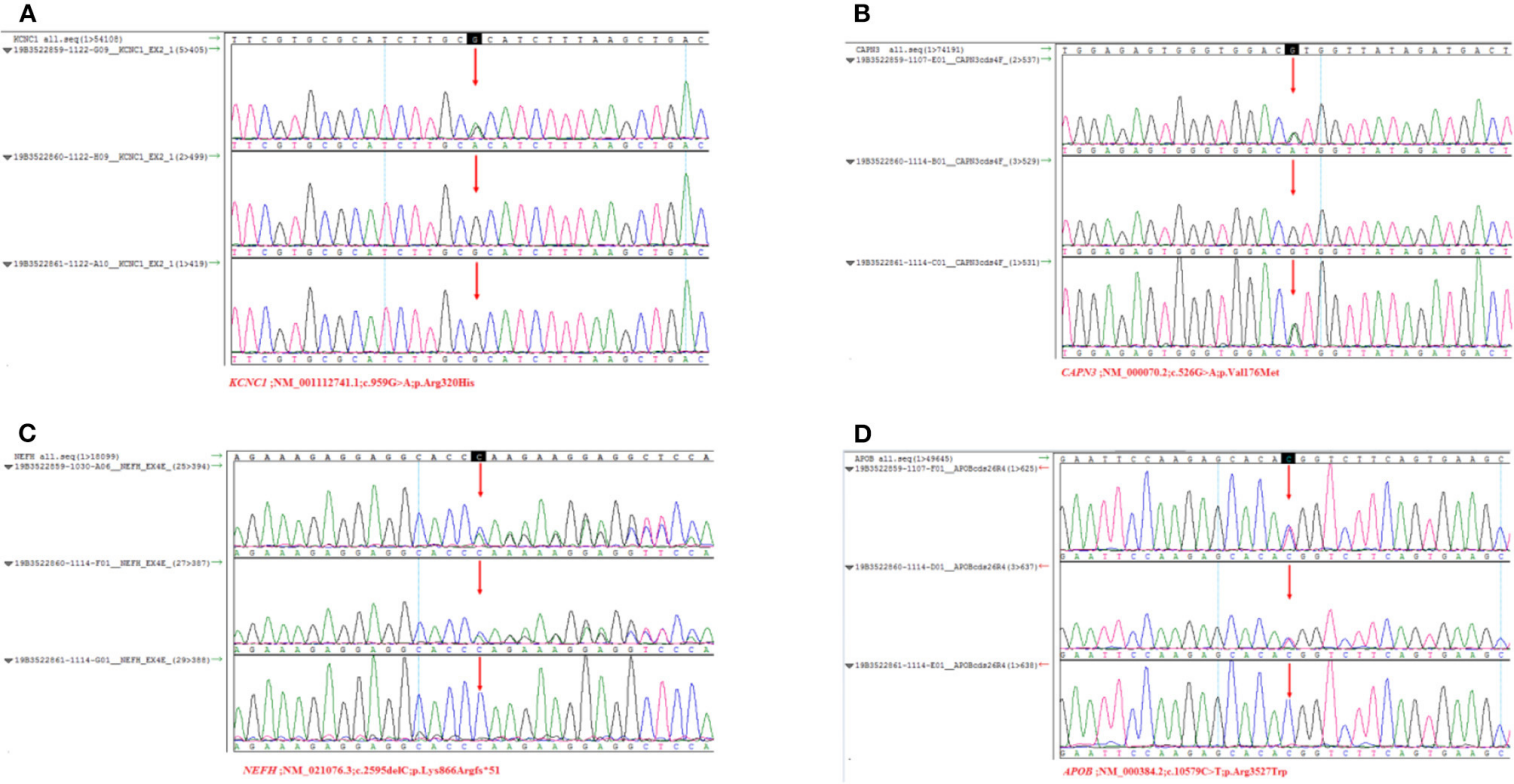
Sanger sequencing results of the *KCNC1, CANP3, NEFH, APOB*. Arrows indicate the verification site of mutation. **(A)** The three results were sequencing results of proband, father and mother. KCNC1 was a new mutation (c. 959G > A, p. Arg320His). There was no heterozygous mutation of variant. **(B)** CAPN3 (c.526G > A, p. Val176Met) at this site, which was inherited from the father. **(C)** NEFH is a heterozygous mutation (c.259 5delC, p. Lys866Arg fs*51) inherited from the mother. **(D)** APOB belongs to heterozygous mutation (c.10579C > T, p. Arg3527Trp), inherited from its mother.

Sanger sequencing confirmed that the c.959G>A mutation in the KCNC1 gene was a *de novo* mutation. The heterozygous mutation *CAPN3* c.526G > A (p. Val176Met) was detected in the proband and his father. The heterozygous variants c.2595delC (p. Lys866Argfs*51) and c.10579C > T (p. Arg3527Trp) detected in the *NEFH* and *APOB* genes of the patient were inherited from his mother. The patient's parents did not report any history of seizures, myasthenia, or myoclonus problems (Figure 1).

### Protein–Protein Interaction Analysis

**Figure 2:**
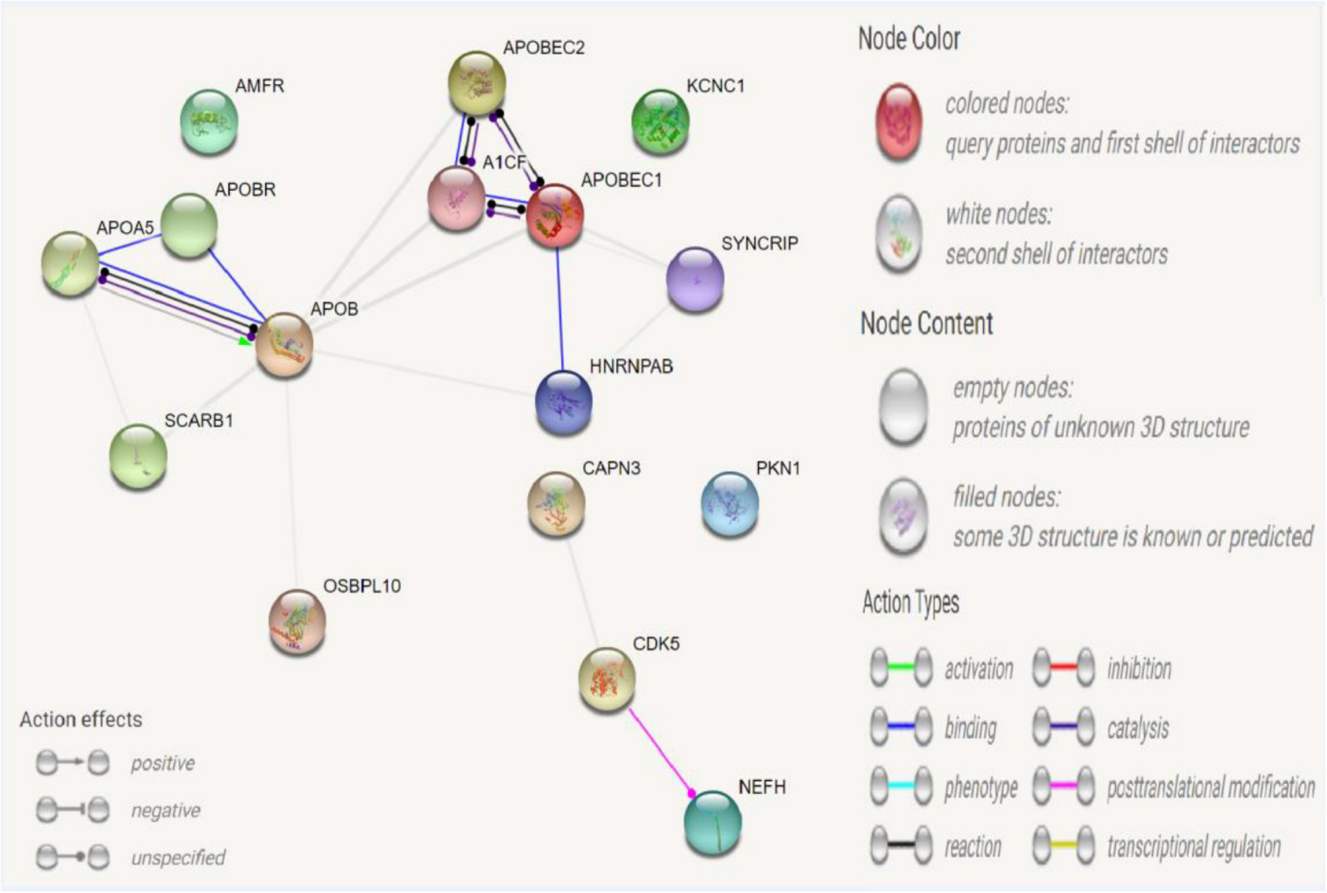
Network for *KCNC1, CAPN3, NEFH, APOB*, and their interacted genes. Network nodes represent proteins. Splice isoforms or post-translational modifications are collapsed, i.e., each node represents all the proteins produced by a single, protein-coding gene locus. Edges represent protein-protein associations: associations are meant to be specific and meaningful, i.e., proteins jointly contribute to a shared function; this does not necessarily mean they are physically binding each other.

**Figure 3:**
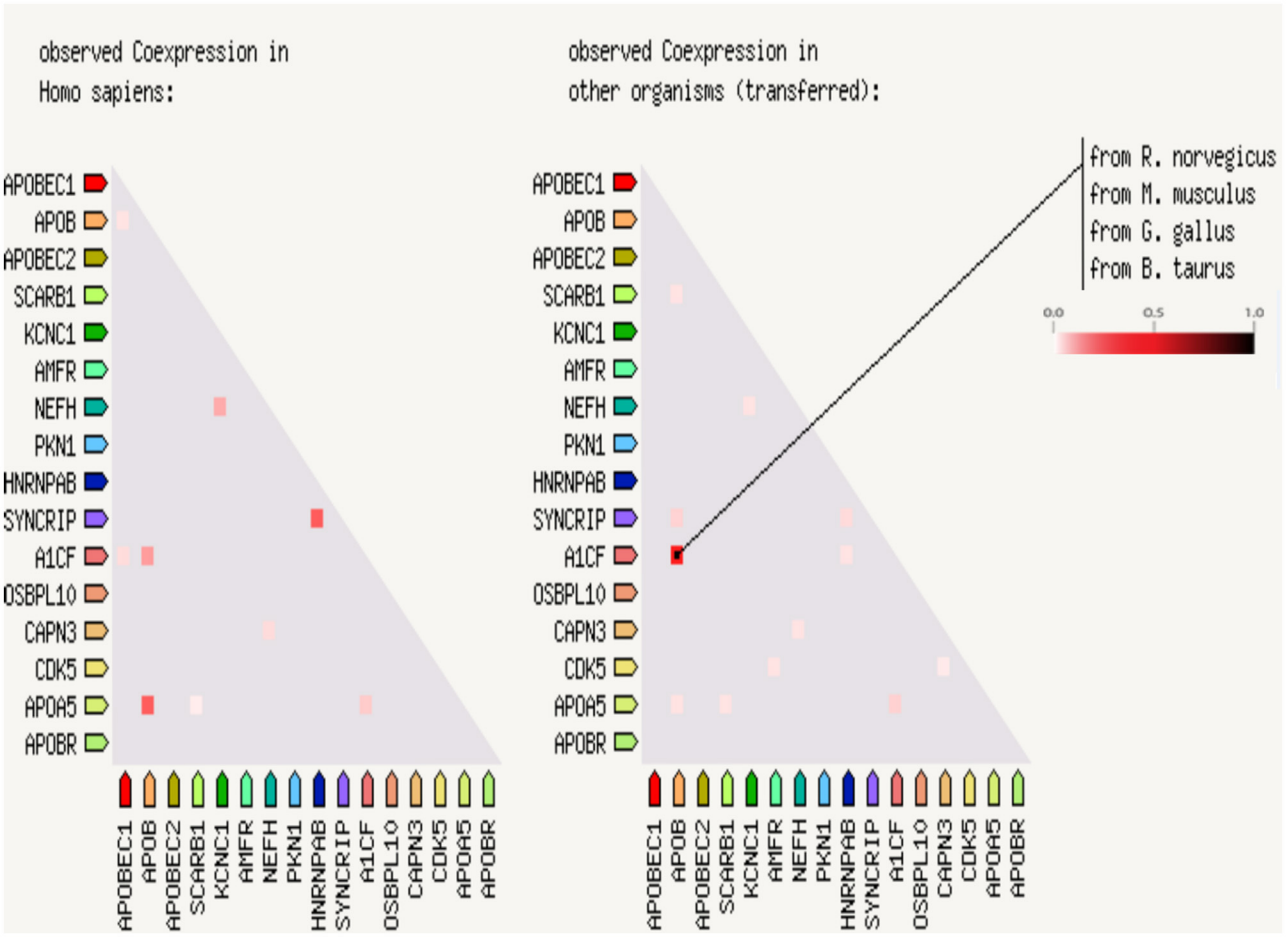
Coexpression scores based on RNA expression patterns and protein co-regulation. In the triangle-matrices above, the intensity of color indicates the level of confidence that two proteins are functionally associated, given the overall expression data in the Homo sapiences and other organisms.

A protein–protein interaction analysis was performed on the following targeted genes and their interacting genes: *KCNC1, CAPN3, NEFH* (interacting genes: *PKN1, CDK5*), and *APOB* (interacting genes: *SYNCRIP, A1CF, APOBR, APOBEC1, APOBEC2, SCARB1, AMFR, HNRNPAB, OSBPL10, APOA5*); the results indicated that the proteins were highly interacting (Figures 2, 3), suggesting that the combination of heterozygous mutations in multiple genes led to a severe phenotype.

## Discussion

Significant advances in the understanding of the neurobiology of seizures and epileptic diseases have resulted in multiple etiologic categories for epilepsy classification. An initial investigation often involves neuroimaging, ideally MRI, where available. The clinician can thus determine whether there is a structural etiology for the patient's epilepsy. The five etiologic groups are genetic, infectious, metabolic, immune, and unknown (2). Therefore, elucidation of the etiology and pathogenesis of seizures is very important for genetic counseling, diagnosis, and effective treatment of seizures. Recent studies on the etiology of epilepsy have shown that genetic factors play an important role in the pathogenesis of epilepsy (21, 22). The whole-exome sequencing technique uses a specific probe to enrich the DNA of the protein-coding regions and detects gene mutations through high-throughput sequencing, which provides good support for the diagnosis of hereditary diseases. Whole-exome sequencing of 10 families of seizures has indicated mutations associated with severe epilepsy and several new disease-causing genes (23). Whole-exome sequencing technology provides new guidance on diseases that are difficult to diagnose only through clinical characterization and laboratory tests, as well as facilitating the diagnosis of diseases and an analysis of etiology and pathogenesis (24). In this study, we found that the presence of *KCNC1, CAPN3*, and *NEFH* mutations in probands was closely related to clinical manifestations such as seizures and muscle weakness. Among these mutations, *KCNC1* was a *de novo* mutation, and the latter two mutations were inherited from the proband's father and mother.

*KCNC1* belongs to the gene family related to potassium-ion channels that are widely distributed in the central nervous system. This gene plays an important role in regulating a series of physiological activities, such as action potential formation, membrane repolarization, and creating tension in muscle cells. Voltage-gated potassium channels are critically important for the rapid repolarization of fast-discharging brain neurons, where Kv3 is a voltage-gated potassium channel consisting of six transmembrane segments (25). The Kv3 subfamily consists of four genes, Kv3.1, Kv3.2, Kv3.3, and Kv3.4. *KCNC1* encodes Kv3.1 and can activate the high-frequency discharge of cells, which is the main determinant of the discharge of high-frequency neurons and facilitates regulation of the excitability of cells (26). *KCNC1* is a highly conserved potassium channel subunit of the voltage-gated tetrameric potassium channel Kv3 subfamily. The Kv3 subfamily is closely associated with neurological disorders, such as *KCNC 3* (Kv3.3) missense mutations found in spinocerebellar ataxia (27) and the absence of KCNC2 chromosomes in patients with neurodevelopmental retardation and ataxia (28). Knocking out *KCNC1* in *KCNC1*-mutant mice has been reported to lead to myopathy and ataxia (29–31). Recently, a new mutation c.959G>A (p. (Arg320His) in *KCNC 1* was identified as a major cause of progressive myoclonic epilepsy (32, 33). Since 2015, exome sequencing has led to the identification of more than 24 *KCNC1* c.959G>A mutation cases in unrelated families (Tables 2, 3). Table 1 shows a list of variants, including those found in our patient and all cases reported thus far, as well as the major clinical symptoms observed in patients with this *KCNC1* c.959G>A mutation. A similar clinical phenotype and findings were obtained for our patients as for most reported patients reported thus far, and 10 patients manifested trembling (Table 3). Being positively charged, the arginine residue contributes to the gated charge (37, 38). This mutation has a negative effect on the Kv3.1 channel by producing electrophysiological abnormalities, resulting in the expression of the corresponding neuron (39). The clinical manifestations reported in previous studies were similar to those of the patient of this study.

**Table 2:** Clinical phenotype of patients with mutation site of c.959G>A.

Gender (male, female)	50.0% male
	50.0% female (12:12)
Initial symptom	
MS	52.2% (12/23)
GTCS	17.4% (4/23)
Trembling	30.4% (7/23)
Ataxic	8.7% (2/23)
FS	4.3% (1/23)
Seizure types	
GTCS	91.3% (21/23)
FS	4.3% (1/23)
MS	95.7% (22/23)
GTCS, MS	87.0% (20/23)
Ataxia	100.0% (24/24)
Mental retardation	63.6% (14/22)

*MS, myoclonic seizure; FS, Focal seizure; GTCS, generalized tonic clonic seizure*.

**Table 3:** The clinical features and gene test result of 24 patients with mutation site of c.959G>A.

**Number**	**Gender**	**Age at onset**	**Initial symptom**	**Seizure types**	**Ataxia**	**Mental retardation**	**Gene mutation site**	**Amino acid variation**	**Age, outcome**	**References**
1	F	10y	MS, GTCS	MS, GTCS	Yes	No	c.959G>A	P.Arg320His	13y; Unsteady walking	(34)
2	F	11y	MS, FS	MS, FS	Yes	Yes	c.959G>A	P.Arg320His	12y; Unsteady walking	(34, 35)
3	M	12y	MS	MS, GTCS	Yes	Yes	c.959G>A	P.Arg320His	38y; He was in a wheelchair at the age of 27	(33, 36)
4	M	6y	MS	MS, GTCS	Yes	Yes	c.959G>A	P.Arg320His	34y; He was in a wheelchair at the age of 17	(33, 36)
5	M	<5y	Ataxic	MS, GTCS	Yes	No	c.959G>A	P.Arg320His	40y; He was in a wheelchair at the age of 16	(33, 36)
6	F	10y	MS	MS, GTCS	Yes	Yes	c.959G>A	P.Arg320His	36y; He was in a wheelchair at the age of 15	(33, 36)
7	M	9y	Trembling, MS	MS, GTCS	Yes	No	c.959G>A	P.Arg320His	24y; Unsteady walking	(33, 36)
8	F	7y	Trembling	MS, GTCS	Yes	No	c.959G>A	P.Arg320His	22y; He was in a wheelchair at the age of 14	(33, 36)
9	F	10y	MS	MS, GTCS	Yes	No	c.959G>A	P.Arg320His	19y; He was in a wheelchair at the age of 17	(33, 36)
10	F	12y	MS	MS, GTCS	Yes	Yes	c.959G>A	P.Arg320His	24y; He can walk at the age of 17	(33, 36)
11	F	9y	MS	MS, GTCS	Yes	Yes	c.959G>A	P.Arg320His	15y; He was in a wheelchair at the age of 13	(33, 36)
12	F	9y	MS	MS, GTCS	Yes	Yes	c.959G>A	P.Arg320His	25y; He was in a wheelchair at the age of 19	(33, 36)
13	F	10y	Trembling	MS, GTCS	Yes	No	c.959G>A	P.Arg320His	42y; Unsteady walking	(33, 36)
14	F	13y	MS	MS, GTCS	Yes	Yes	c.959G>A	P.Arg320His	37y; Unsteady walking	(33, 36)
15	M	12y	Ataxic	MS, GTCS	Yes	Yes	c.959G>A	P.Arg320His	19y; Unsteady walking	(33, 36)
16	F	14y	GTCS	GTCS	Yes	No	c.959G>A	P.Arg320His	16y; Unsteady walking	(33, 36)
17	M	10y	MS	MS, GTCS	Yes	Yes	c.959G>A	P.Arg320His	18y; Unsteady walking	(33, 36)
18	M	NA	NA	NA	Yes	NA	c.959G>A	P.Arg320His	NA	(33, 36)
19	M	8y	Trembling, MS	MS	Yes	No	c.959G>A	P.Arg320His	18y; Unsteady walking	(36)
20	M	12y	GTCS	MS, GTCS	Yes	Yes	c.959G>A	P.Arg320His	17y;	(36)
21	F	15y	Trembling, MS	MS, GTCS	Yes	Yes	c.959G>A	P.Arg320His	40y; Unsteady walking	(36)
22	M	9y	Trembling, MS	MS, GTCS	Yes	Yes	c.959G>A	P.Arg320His	63y; Die of pneumonia and respiratory failure	(36)
23	M	9y	Trembling	MS, GTCS	Yes	Yes	c.959G>A	P.Arg320His	12y; Hypophrenia, Ataxic	(32)
24	M	7y	GTCS	MS, GTCS	Yes	NA	c.959G>A	p.Arg320His	10y; Myoclonics	(32)

*NA, not available; MS, myoclonic seizure; FS, Focal seizure; GTCS, generalized tonic clonic seizure*.

*CAPN3* encodes a muscle-specific member of the calcium-activated neutral protease family. Mutations in the *CAPN 3* gene are associated with the pathogenesis of limb muscular dystrophy type 2A (*LGMD2A*) and limb-girdle muscular dystrophy II. In many patients with myopathy, dozens of different *CANP3* gene mutations have been detected. The main clinical manifestations are progressive symmetrical muscle atrophy and proximal limb muscle weakness (40). A novel homozygous missense mutation of the *CAPN3* gene (c.1699G > A) was detected in a Saudi Arabian family (41). Studies have suggested that a pair of calpain protein lesions carry the mutations c.146G>A and c.329G>A in the CANP3 gene, and the clinical manifestation is LGMD2A (limb muscular dystrophy type 2A). The mutation site of the former is located in the short N-terminal domain 1 of the calpain-3 protein, which encodes a regulatory propeptide rich in cysteinase (42), whereas the mutation of the latter encodes the IIa domain of the calpain-3 protein, which forms a catalytic crack related to self-degradation together with the IIb domain (40). *CAPN3* mutation can be considered a powerful genetic factor leading to LGMD2A disease. Previous studies have found that HSD17B10 C.526G>A leads to problems of kinetic damage and complex formation (43). According to the ACMG guidelines, the variation is suspected to be pathogenic. In this study, a novel variant C.526G > A (p. Val176Met) was identified that has not previously been reported in patients. This variant is a heterozygous mutation that the patient inherited from his father, although both patient's parents had no related clinical manifestations. Similar manifestations seem to exist in this patient, such as unsteady walking and ease of falling.

*NEFH* is the pathogenic gene of Charcot-Marie-Tooth 2 (CMT2), and the hereditary model is autosomal dominant inheritance. Charcot-Marie-Tooth disease is a disease of the peripheral nervous system that is characterized by progressive muscle weakness and muscle atrophy. The clinical manifestation of *CMT2* is axonal degeneration without myelinated lesions, and the nervous transmission rate is normal or slightly decreased. *CMT2* can lead to progressive distal muscle weakness and atrophy, which is partly consistent with the clinical manifestations observed in this study. Whole-exome sequencing revealed a mutation (NEFH c.2595delC, p. Lys866Argfs*51). There have been no reports on the pathogenicity of this mutation. It has been reported that in the CMT family, *NEFH* mutations interfere with neurofilament assembly by protein sequestration and cause neurotoxicity (44, 45). In the present study, Sanger sequencing confirmed that the variation in *NEFH* was inherited from the mother. The patient had clinical manifestations, such as unstable gait, but the parents had no relevant clinical manifestations.

*APOB* apolipoprotein E (apo E) is a 34 kDa glycosylation and excretion protein, and APOB is a major protein constituent of chylomicrons (apo B-48), LDL (apo B-100), and VLDL (apo B-100). *APOB* is associated with familial hypercholesterol type 1 and autosomal dominant hypercholesterolemia type B. An *APOB* mutation (c.10579C>T, p. Arg3527Trp) was detected by unexpected detection and reported as a pathogenic mutation based on ACMG guidelines. The respective patient was found to have an *APOE* mutation (p. Leu167del), which was the cause of dominant inheritance of familial hypercholesterolemia (20, 46).

Epilepsy is a heterogeneous disease characterized by abnormal signal transduction of neurotransmitters and abnormal ion channels. The etiology of epilepsy is complex, and many studies have shown that genetic factors are the main cause of epilepsy. The clinical manifestations of epilepsy are phenotypically heterogeneous; that is, a gene associated with epilepsy may have different clinical characteristics. In this study, three genes, *KCNC1, CAPN3*, and *NEFH*, were found to be genetically mutated. The former is a *de novo* mutation, and the latter two heterozygous mutations were inherited from the proband's father and mother. *CANP3* and *NEFH* are suspected pathogenic genes closely related to the clinical manifestations of patients. The STRING database (http://string-db.org) provides a critical assessment and integration of protein–protein interactions, including direct (physical) and indirect (functional) associations, and was used to analyse the evidence for the interaction of *KCNC1, CAPN3, NEFH*, and *APOE*. It seems that the heterogeneity of patient phenotypes results from the interaction of multiple mutated genes. Although our study identified *KCNC1* as the main cause of pathogenic mutations, the role of *NEFH* and *CAPN3* in mutations may be a fortuitous phenomenon. However, we should not ignore the multigene interactions leading to the diversity of epilepsy phenotypes in clinical analysis. Meanwhile, in this case, due to the application of gene sequencing, the disease of the proband was reasonably explained, which laid a foundation for finding a better treatment plan and brought the possibility of healthy growth and development of the next generation for similar families.

With the rapid development of gene sequencing technology, especially the application of whole-exome sequencing technology, the problems of case divergence, gene-site heterogeneity and exon incompleteness in the diagnosis of epilepsy and other diseases have been solved. Whole-exome technology has high sensitivity and accuracy and can be used to identify rare monogenic genetic diseases, which considerably facilitates the study of the etiology of diseases. Whole-exome sequencing technology is used to capture epilepsy-related pathogenic genes and their mutation sites, after which prenatal diagnosis can be performed by Sanger sequencing technology to detect the causes of rare clinical diseases and carry out genetic diagnosis for high-risk groups with a family history of disease. This technology is a reliable means of identifying pathogenic genes and exploring pathogenic mechanisms that can be employed in prenatal diagnosis and genetic counseling to effectively reduce the risk of having children with epilepsy. Thanks to gene sequencing technology, we can diagnose, treat, and follow up patients with genetic abnormalities as the main line. Overall, based on the previous information on gene mutations, we could not make a definite connection on these genes, but analysis of the genotype and phenotype correlation of this case alone does not rule out the possibility.

## Data Availability Statement

The datasets presented in this study can be found in online repositories. The names of the repository/repositories and accession number(s) can be found below: SAMN21236071 (https://www.ncbi.nlm.nih.gov/biosample/?term=Epilepsy-FJS-%2001).

## Ethics Statement

The studies involving human participants were reviewed and approved by the IRB at the Third Affiliated Hospital of Guangzhou Medical University (No. 2021-113). The patients/participants provided their written informed consent to participate in this study.

## Author Contributions

XY designed the study. WQ, LZ, WD, and SX performed the experiments. HQ and ZJ wrote the paper. All the authors read and approved the final manuscript.

## Funding

This study was supported by the National Natural Science Foundation of Guangdong Province (Grant No. 2020A0505100062), Clinical Innovation Research Program of Guangzhou Regenerative Medicine and Health Guangdong Laboratory (Grant No. 2018GZR0201002), and 2018 Dr. Start Research Fund of the Third Affiliated Hospital of Guangzhou Medical University (Grant No. 2018B11).

## Conflict of Interest

The authors declare that the research was conducted in the absence of any commercial or financial relationships that could be construed as a potential conflict of interest.

## Publisher's Note

All claims expressed in this article are solely those of the authors and do not necessarily represent those of their affiliated organizations, or those of the publisher, the editors and the reviewers. Any product that may be evaluated in this article, or claim that may be made by its manufacturer, is not guaranteed or endorsed by the publisher.
